# Highly sensitive and specific bioassay for measuring bioactive TGF-β

**DOI:** 10.1186/1471-2121-7-15

**Published:** 2006-03-20

**Authors:** Ina Tesseur, Kun Zou, Elisabeth Berber, Hui Zhang, Tony Wyss-Coray

**Affiliations:** 1 Department of Neurology and Neurological Sciences, Stanford University School of Medicine, Stanford, CA 94305, USA; 2 GRECC, VA Palo Alto Health Care System, Palo Alto, CA 94304, USA; 3 Department of Alzheimer's Disease Research, National Center for Geriatrics and Gerontology, Obu, Aichi 474-8522, Japan

## Abstract

**Background:**

Transforming Growth Factor-β (TGF-β) regulates key biological processes during development and in adult tissues and has been implicated in many diseases. To study the biological functions of TGF-β, sensitive, specific, and convenient bioassays are necessary. Here we describe a new cell-based bioassay that fulfills these requirements.

**Results:**

Embryonic fibroblasts from *Tgfb1*^-/- ^mice were stably transfected with a reporter plasmid consisting of TGF-β responsive Smad-binding elements coupled to a secreted alkaline phosphatase reporter gene (SBE-SEAP). Clone MFB-F11 showed more than 1000-fold induction after stimulation with 1 ng/ml TGF-β1, and detected as little as 1 pg/ml TGF-β1. MFB-F11 cells were highly induced by TGF-β1, TGF-β2 and TGF-β3, but did not show induction with related family members activin, nodal, BMP-2 and BMP-6 or with trophic factors bFGF and BDNF. MFB-F11 cells can detect and quantify TGF-β in biological samples without prior enrichment of TGF-βs, and can detect biologically activated TGF-β in a cell co-culture system.

**Conclusion:**

MFB-F11 cells can be used to rapidly and specifically measure TGF-β with high sensitivity.

## Background

Transforming growth factor-β (TGF-β) is the prototype member of a superfamily of growth factors that includes activins, bone morphogenic proteins (BMPs), inhibins, growth and differentiation factors (GDFs), and glial derived neurotrophic factor (GDNF) [1]. TGF-β is widely distributed in both embryonic and adult tissues and regulates cell proliferation and differentiation, extracellular matrix production, wound healing, immune function, apoptosis, angiogenesis, chemotaxis and hematopoiesis. Many human diseases, including certain forms of cancer as well as fibrotic and inflammatory disorders, have alterations in the TGF-β signaling pathway (reviewed in [2,3]). Recently, TGF-β has also been implicated in Alzheimer's Disease and other neurodegenerative conditions [4,5].

In mammals, TGF-β has three closely related isoforms, TGF-β1, -2 and -3, which share 70% sequence identity but exhibit different functions *in vivo *(reviewed in [1]). The biological functions of TGF-β are mediated through a high-affinity transmembrane receptor complex consisting of TGF-β type I (ALK5) and type II serine/threonine kinase receptor subunits (reviewed in [1,6,7]). Upon binding of the ligand, ALK5 recruits and phosphorylates receptor-regulated Smad2 or Smad3, which then form heteromeric complexes with Smad4 and translocate into the nucleus, where together with other transcription factors they regulate gene transcription [6]. Smad3 binds directly to DNA via a conserved Smad-binding element (SBE) that consists of one or more CAGA boxes [8]. This CAGA box is present in an estimated 500 target genes for TGF-β in mammals [6,9]. Smad2 normally does not bind to DNA, although an alternatively spliced form of Smad2, which lacks exon 3, can bind with high affinity to SBE and appears to be present in many cell types [10]. Activins, nodal, growth and differentiation factor (GDF)-8/myostatin, GDF-9, and GDF-11 also signal via Smad2/3 proteins by engaging activin receptors or ALK5 [6,11-14]. In contrast, BMPs recruit Smad1, Smad5, and Smad8 in combination with Smad4 after binding to BMP type I and type II receptors [6,7]. In addition, signaling of TGF-β s and related factors is regulated by crosstalk with other signaling pathways including MAP kinase, JAK/STAT, and Wnt pathways (reviewed in[15]).

TGF-β is secreted as a latent pro-peptide, and does not signal unless activated. Activation *in vivo *is still poorly understood and can involve integrins, thrombospondin, metalloproteases, plasmin, furin and other proteases [16,17]. Bioactive TGF-β peptides are rapidly inactivated in living cells and consequently, bioactive TGF-β peptides are usually not detectable in tissues, body fluids, or supernatants from cultured cells. However, latent TGF-β in such samples can be "activated" with acids, heat, or other methods [18]. To measure levels of bioactive TGF-β in activated samples, several assays including wound healing and growth inhibition assays or reporter gene assays have been developed [18-21], but many of these assays are cumbersome and complicated or can be influenced by other growth factors present in the samples. Currently one of the most frequently used bioassays is based on a mink lung cell line (TMLC) stably transfected with a plasminogen activator inhibitor-1 (PAI-1) promoter fused to luciferase [19]. TMLC cells have also been used in coculture with other cells to measure their TGF-β production [22,23]. In this paradigm, TMLC cells seem to measure TGF-β activated via integrins [22]. A disadvantage of the TMLC cells however, is that they can be induced by other growth factors present in the samples [19,21].

Here we describe a new bioassay that is specific for TGF-βs, is sensitive and reproducible and allows for repeated measurements of the same sample in induced reporter cells. We stably transfected mouse embryonic fibroblasts from *Tgfb1*^-/- ^mice with a synthetic promoter element containing twelve CAGA boxes [8], fused to a secreted alkaline phosphatase (SEAP) reporter gene. Clone MFB-F11 showed up to 1000 fold induction with 1 ng/ml TGF-β1 (40 pM) and showed a linear response to TGF-β1 from 1 pg/ml to 10 ng/ml. TGF-β1, TGF-β2 and TGF-β3 but not other related TGF-β family members activated the reporter gene in these cells. We demonstrate that this new cell line can be used to quantitatively measure TGF-β bioactivity from body fluids, tissues and cell culture supernatants. Importantly, MFB-F11 cells can detect TGF-β bioactivity directly from other cells in co-culture systems.

## Results

### Generation of SBE-SEAP reporter cells to measure TGF-β signaling

To measure TGF-β bioactivity from cell culture supernatants or biological fluids rapidly, specifically, and with high sensitivity we developed a new cell-based reporter assay. We used a fibroblast cell line isolated from mouse *Tgfb1*^-/- ^embryos (MFB), which showed low background and high induction of the SBE-SEAP reporter during transient transfections. The SBE-SEAP reporter plasmid was generated from a previously described SBE-luciferase plasmid [8] by replacing firefly luciferase with SEAP. The SEAP protein is secreted into the medium, and allows multiple measurements from the same culture.

**Figure 1:**
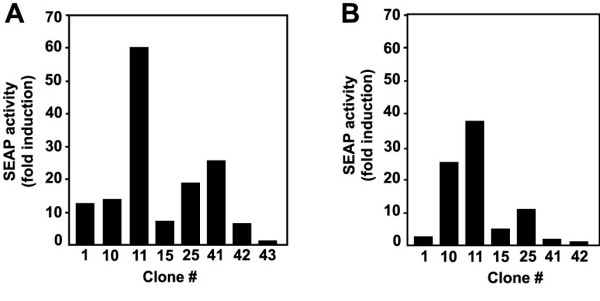
Selection of MBF-F11 cells. *Tgf-b1*^-/- ^mouse embryonic fibroblast clones that are stably transfected with SBE-SEAP (Clones 1 – 42) show induction of the reporter after treatment with 1 ng/ml TGF-β1 at passage 2 (A) and at passage 9–12 (B). Clone 43 is a control non-inducible clone. Assays were performed in 1 ml total volume and SEAP activity was measured in 10 μl supernatant after 24 h induction. The baseline measurement (no TGF-β) of each clone was used to calculate fold induction.

MFB cells were stably transfected with SBE-SEAP and a total of 49 stably-transfected colonies were examined for induction of reporter activity by TGF-β1. Out of 7 clones with strong induction, clone MFB-F11 showed the highest and most consistent activity with relatively low background levels (Figure 1A). Clones that showed induction at passage 2 were retested after 9–12 passages (Figure 1B). Clone MFB-F11 was not only the most inducible but also the most stable clone, and was still inducible without significant loss of sensitivity after more than 30 passages (data not shown).

### MFB-F11 cells can measure as low as 1 pg/ml TGF-β1

**Figure 2:**
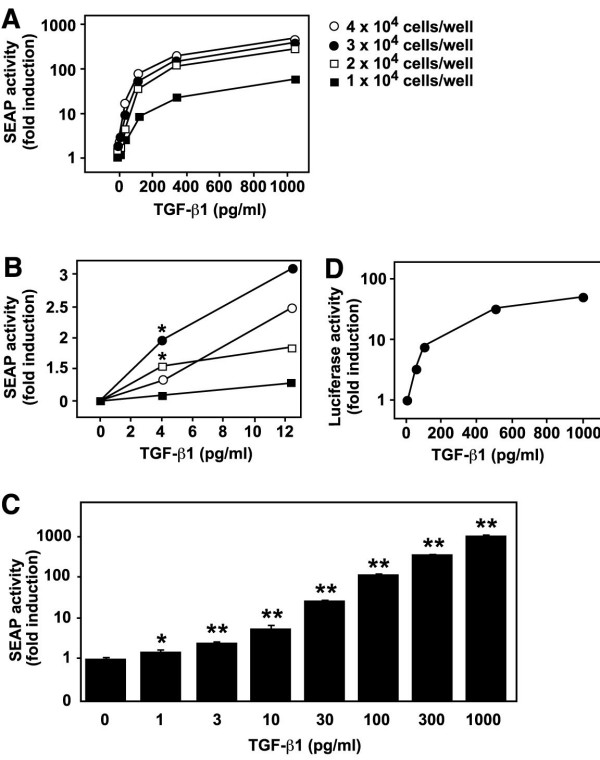
TGF-β1 dose-dependently induces SEAP reporter activity in MFB-F11 cells. A, B: MFB-F11 cells were cultured at different cell densities with the indicated concentrations of TGF-β1 and SEAP activity was measured in the supernatant. (B) shows a higher magnification of the x-axis shown in (A) in the lower range of TGF-β1. C: 3 × 10^4 ^MFB-F11 cells/well were incubated with the indicated concentrations of TGF-β1 in B27 supplemented, serum-free DMEM. Note the logarithmic scale. Bars represent mean ± SEM of triplicate wells from one representative experiment. D: 4 × 10^4 ^TMLC cells/well were incubated with the indicated concentrations of TGF-β1 in serum-free DMEM. All assays were performed in 100 μl total volume in 96-well tissue culture plates and SEAP or luciferase activity was measured after 24 h induction in 10 μl supernatant or 50 μl cell pellet respectively. * *P *< 0.05; ** *P *< 0.001, Student's *t *test compared to 0 pg/ml TGF-β1 in C.

To further characterize MFB-F11 cells, we assayed induction of reporter activity using a broad range of TGF-β1 concentrations at different cell densities. Higher cell densities showed a better induction with TGF-β1 concentrations in the ng/ml range (Figure 2A), whereas 30,000 cells/well in a 96-well tissue culture plate seemed to work best with TGF-β1 concentrations between 1 and 100 pg/ml (Figure 2B). At 30,000 cells/well MFB-F11 showed a significant 2-fold induction with only 1 pg/ml TGF-β1 in the presence of B27 supplement, which provides the cells with additional nutrients when cultured in serum free medium (Figure 2C, *P *< 0.05 unpaired Student's *t *test), and the response was linear up to 1 ng/ml TGF-β1. At this concentration the reporter was induced more than 1000 fold (Figure 2C). Higher cell densities did not further increase induction and usually resulted in lower induction (data not shown). TMLC cells, which have been widely used to measure TGF-β activity in cell culture, showed a similar dose-dependent response to TGF-β1. In our hands however, the maximal fold induction was higher in MFB-F11 cells (Figure 2A and 2D).

### MFB-F11 cells specifically measure TGF-β1, TGF-β2 and TGF-β3

**Figure 3:**
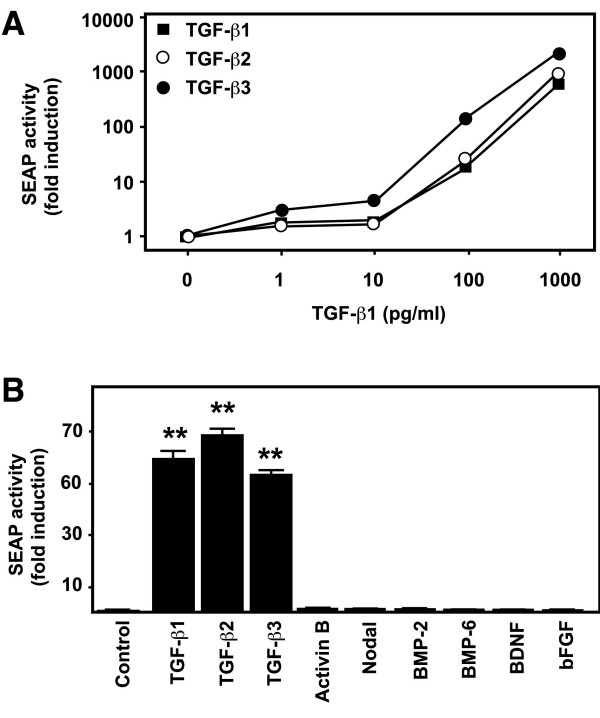
MFB-F11 cells are specific for TGF-βs. A: MFB-F11 cells (4 × 10^4^/well) were stimulated with the indicated concentrations of TGF-β1, 2 and 3. Note the logarithmic scale. B: MFB-F11 cells (4 × 10^4^/well) were stimulated with 0.1 ng (TGF-β1, 2, 3) or 10 ng (activin B, nodal, BMP2, BMP6, BDNF, bFGF) of growth factors. SEAP activity was measured after 24 h as described in Figure 2. Bars represent mean ± SEM of triplicate wells from 1 representative experiment. ** *P *< 0.001, Student's *t *test compared to control.

To examine whether MFB-F11 cells would also respond to growth factors other than TGF-β1, we tested induction of SEAP activity with TGF-β2, TGF-β3, nodal, activin B, BMP2, BMP6, GDNF and bFGF. The closely related TGF-β1 family members TGF-β2 and TGF-β3 induced SEAP activity in MFB-F11 cells in a dose-dependent manner (Figure 3A). In contrast, nodal, activin B, BMP2, BMP6, BDNF and bFGF did not induce SEAP acitivity, even at high concentrations (Figure 3B) or after 48 or 96 h induction (data not shown). The latter also did not induce SEAP reporter activity when added together with different amounts of recombinant TGF-β1, TGF-β2 or TGF-β3 (data not shown). Taken together these results suggest that MFB-F11 cells respond specifically to TGF-βs.

### MFB-F11cells measure TGF-βs from biological samples

**Figure 4:**
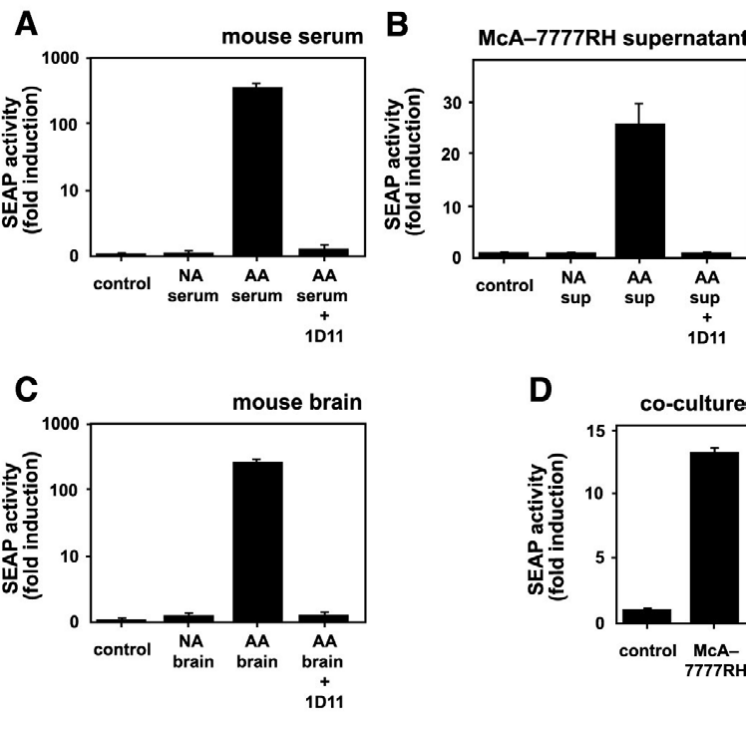
MFB-F11 cells can measure biologically active TGF-β and TGF-βs from various sources and species. MFB-F11 cells (4 × 10^4 ^cells/well) were incubated with 100 μl diluted (1: 100) mouse serum (A), McA-7777RH cell culture supernatant (B) or diluted (1:50) mouse brain extract (C) which were either not activated (NA) or activated with acid (AA). Activated medium was incubated with a pan-specific TGF-β neutralizing antibody (AA + 1D11) or left untreated (AA). D: 4 × 10^4 ^MFB-F11 cells/well were left untreated or co-cultured with 4 × 10^4 ^McA-7777RH cells/well. SEAP activity was measured after 24 h as described in Figure 2. Bars represent mean ± SEM of triplicate wells from 1 representative experiment. Note the logarithmic scale in A and C.

To determine the use of MFB-F11 cells in measuring TGF-β from biological samples, we tested mouse serum, mouse brain extract and conditioned medium from McA-7777RH liver cells (Figure 4A–C) or Chinese hamster ovary (CHO) cells (data not shown). After activation of presumably latent TGF-β with acid all samples were able to strongly induce reporter activity in MFB-F11 cells (Figure 4A–C). Co-incubation of activated samples with a TGF-β neutralizing antibody nearly completely inhibited induction (Figure 4A–C), demonstrating that MFB-F11 cells specifically measure TGF-β activity in biological samples. Samples that were not activated with acid did not induce reporter activity (Figure 4A and 4B) consistent with the presence of TGF-β in a latent form in biological samples [16].

To determine the use of MFB-F11 cells in measuring biologically activated TGF-β, we co-cultured them with other cell types. Co-culture with McA-7777RH cells (Figure 4D) or B103 cells (data not shown) resulted in significant induction of SEAP activity. The relative activity detected in this co-culture system was lower than what was measured in acid activated conditioned medium of the same cells (Figure 4B), suggesting that part of the secreted TGF-β is in a latent form and cannot be detected by MFB-F11 cells.

Taken together these data demonstrate that MFB-F11 cells can be used to specifically measure TGF-β1, TGF-β2 and TGF-β3 activities with high sensitivity in biological samples.

## Discussion

In this study we describe the generation of a stably transfected *Tgf-b1*^-/- ^fibroblast cell line (clone MFB-F11), which provides a specific, sensitive, reproducible and convenient bioassay for the quantification of TGF-βs in biological samples from different species.

The major advantages of the presented method over previous TGF-β bioassays are the high sensitivity and specificity of MFB-F11 cells for TGF-βs. MFB-F11 cells can reliably measure as little as 1 pg/ml TGF-β1 and their linear range extends well beyond 1000 pg/ml (Figure 2), whereas a typical TGF-β1 ELISA measures between 32–1000 pg/ml TGF-β1 [24]. Although the MFB-F11 assay and TMLC luciferase assay measure TGF-β1 with the same sensitivity at the lower end [19], the fold induction of the MFB-F11 assay compare favorably to the TMLC luciferase assay. This increased dynamic range and higher fold induction could be the result of an increased stability or accumulation of the SEAP protein in the medium. Another disadvantage of the luciferase assay is that it requires lysis and harvesting of cells, whereas the SEAP assay only requires 10 μl of cell culture medium to measure induction. As removal of a small part of cell culture medium is non invasive, it also allows sequential measurements of induction in the same cells. In addition, the cell pellet can be used to measure other biochemical parameters.

The second advantage of our assay is the specificity of MFB-F11 cells for TGF-βs. Smad proteins play a key role in the intracellular signaling of TGF-βs and its closely related family members. Smad2 and Smad3 transduce signals from TGF-βs, activins, nodal, inhibins and GDF1 to the nucleus, whereas Smad1 and Smad5 are the intracellular signaling components of BMPs and GDF5 signaling (reviewed in [1,6]). Smad3 and Smad4, but not Smad1 or Smad2 were shown to bind to SBE [8], which was used to generate the SBE-SEAP reporter construct present in the MFB-F11 cells. Interestingly, both nodal and activin do not activate the reporter in the MFB-F11 cells (Figure 2). This is in contrast with signaling in SBE-luciferase primary astrocytes, where both activin and nodal were able to induce luciferase reporter activity [25], and in TMLC cells, where other growth factors were able to induce reporter activity, either alone or in combination with TGF-βs [19,21]. It is possible that MFB-F11 cells lack some of the receptors necessary for activin and nodal signaling. The differences in specificity between the TMLC and MFB-F11 cells could also be due to differences in promoter elements preceding the reporter gene. MFB-F11 cells contain a minimal 12-repeat CAGA box, which has been described to only respond to Smad-mediated signaling [8], whereas the TMLC cells contain a PAI-1 promoter element, which is able to bind additional transcription factors [26]. In any case, both the absence of reporter induction by TGF-β related factors (Figure 2), as well as by biological samples treated with pan-specific TGF-β neutralizing antibodies (Figure 3), demonstrates the high specificity of MFB-F11 cells for TGF-βs. In addition the MFB-F11 bioassay is valid across species and can measure TGF-βs from human (recombinant human TGF-β1, 2 or 3 and TGF-βs present in human serum), mouse, rat (Figure 2, Figure 4) and hamster (data not shown).

The third advantage of our assay is that it can measure biologically active TGF-β secreted by cells (Figure 4D). Most cells secrete TGF-βs as inactive complexes, although freshly isolated LPS-stimulated murine peritoneal macrophages were able to activate TGF-βs [27]. In TMLC cells, co-culture with a different cell type resulted in activation of the reporter when β 6-integrin was overexpressed in the co-cultured cell line [22,23]. In contrast, MFB-F11 cells were able to detect TGF-β activity from two different co-cultured cell lines without further manipulations. It is important to note that induction of SEAP-activity via co-culture was lower than induction via acid-activation of conditioned medium from the same cells (Figure 4B and 4D), indicating that MFB-F11 cells were able to detect only a portion of secreted TGF-βs. The fact that we did not observe SEAP induction in non-acid treated conditioned medium, but only in co-culture suggests that cell-cell contact or close proximity is required for biological activation of secreted TGF-βs. Biologically active TGF-β could be associated with the cell membrane [28-30] or secreted in such low amounts that it needs to be secreted immediately adjacent to the reporter cell to be detectable and secretion into conditioned medium could dilute or inactivate TGF-βs.

## Conclusion

We generated a new bioassay that can measure physiological amounts of TGF-β present in biological samples in a highly sensitive, specific, reproducible, and convenient way without the need for prior enrichment of TGF-βs in the samples. Besides quantifying TGF-β this bioassay can also be used to study TGF-β signaling and to rapidly identify new endogenous or pharmaceutical modifiers of the TGF-β signaling pathway in the absence of TGF-β1.

## Methods

### Cloning of reporter plasmid

A TGF-β responsive NheI/HindIII fragment, containing 12 repeats of the (CAGA) box and a herpes simplex virus thymidine kinase minimal promoter (TK) promoter was isolated from a SBE_12_-luciferase reporter gene ([8], kindly obtained from Dr. D. Vivien, University of Caen, France). This fragment was then ligated into the NheI/HindIII site of the pSEAP2-Basic plasmid (BD Biosciences, San Jose, CA). The final structure of the SBE-SEAP reporter plasmid was confirmed by sequencing.

### Cell lines and generation of reporter cells

Transformed Mink Lung Cells (TMLC) stably transfected with a PAI-1-luciferase reporter gene (obtained from Dr. D. Rifkin; New York University, NY) and McA-RH7777 rat hepatoma cells (ATCC CRL 1601, Manasses, USA) were cultured in DMEM medium supplemented with penicillin/streptomycin and 10% fetal bovine serum (GIBCO, Carlsbad, CA). To generate TGF-β reporter cells, mouse fibroblasts isolated from *Tgfb1*^-/- ^mice (MFB) were stably transfected with the SBE-SEAP plasmid. Briefly, MFB cells were seeded at 10^5 ^cells/well in 6-well plates and transfected 16 h later with 2 μg SBE-SEAP, and 0.2 μg hygromycin resistance plasmid (Promega, Madison, WI) using Lipofectamine Plus (Invitrogen, Carlsbad, CA), according to manufacturer's instructions. Two days later, cells were reseeded at different densities and selected for antibiotic resistance using 100 μg/ml hygromycin B (Invitrogen, Carlsbad, CA). Individual colonies were isolated, expanded, and tested in reporter cell assays.

### Reagents and samples for bioassays

Transforming Growth Factor-β1 (TGF-β1), TGF-β2, TGF-β3, activin B, BMP2, BMP6 and nodal were all purchased from R&D Systems (Minneapolis, MN), basic FGF (bFGF) was from PeproTech, Inc (Rocky Hill, NJ), and GDNF was from Upstate (Charlottesville, VA). All factors were recombinant human proteins. A TGF-β antibody (1D11) neutralizing all three TGF-β isoforms was purchased from R&D systems (Minneapolis, MN). Mouse serum was obtained from NIH wild type mice by cardiac puncture, and spun for 10 minutes at 6000 g. Serum was kept at 4°C until use. Mouse brain was homogenized in homogenization buffer (137 mM NaCl, 20 mM Tris-HCl pH 7.4, 1% NonidetP-40, 10% glycerol and proteinase inhibitors (Roche, Indianapolis, IN)), spun for 20 minutes at 2000 g and supernatants stored at -80°C until use. To produce cell culture supernatants for bioassays, McA-RH7777 rat hepatoma cells were seeded at 10^5 ^cells/well in 12-well plates in serum containing medium, washed with phosphate-buffered saline (PBS) for 16 h and incubated with serum free DMEM for 24 h.

### TGF-β bioassay

MFB-F11 cells were seeded at 1 to 4 × 10^4 ^cells/well in 96-well flat-bottom tissue culture plates (BD Falcon, San Jose, CA). After overnight incubation, cells were washed twice with PBS and incubated in 50 μl serum-free DMEM supplemented with penicillin/streptomycin (DMEM/P/S) for 2 h before recombinant proteins diluted in DMEM/P/S or test samples were added in 50 μl volume. In some experiments B27 supplement (Invitrogen, Carlsbad, CA) was added to serum-free media. Conditioned medium from cells, mouse brain extract (diluted 1:50) or mouse serum (diluted 1:100) was added directly (not activated (NA) condition) or was activated by adding 2.5 μl 6 M HCl to 50 μl sample at room temperature for 10 min followed by neutralization to pH 7.4 with 6 M NaOH (acid activated (AA) condition). In some experiments, TGF-β bioactivity was neutralized before addition to reporter cells by incubating samples with 10 μg/ml of a neutralizing antibody (clone 1D11, R&D Systems, Minneapolis, MN) for 1 h at room temperature. 10 μl aliquots of the culture supernatants were collected after 24 and 48 h incubation with MFB-F11 cells. SEAP activity was measured using Great EscAPe SEAP Reporter system 3 (BD Biosciences, San Jose, CA) according to the manufacturer's instructions with a Lmax plate photometer (Molecular Devices, Sunnyvale, CA). Luciferase activity was detected using the Luciferase assay system from Promega (Madison, WI) and measured with a Lumat LB 9507 tube luminometer (EG&G Berthold).

### TGF-β co-culture bioassay

MFB-F11 cells were seeded at 4 × 10^4 ^cells/well (Invitrogen, Carlsbad, CA) in 96-well flat-bottom tissue culture plates and allowed to attach for 5 – 12 h. Test cells (McA-7777RH or B103) were seeded on top at the same density in DMEM supplemented with 10 % FBS and allowed to attach for another 4 – 5 h. Wells were washed twice with PBS and incubated with 100 μl serum free DMEM. SEAP activity was measured in 10 μl supernatant 24 h later.

## List of abbreviations

ALK5: activin like receptor kinase 5; BDNF: brain derived neurotrophic factor; BMP: bone morphogenic protein; DMEM: dulbecco's modified eagle's medium; FGF: fibroblast growth factor; GDF: growth and differentiation factor; GDNF: glial derived neurotrophic factor; JAK/STAT: janus kinase/signal transducers and activators of transcription; MAPK: mitogen activated protein kinase; MFB-F11: mouse *Tgfb1*^-/- ^embryonic fibroblast clone F11; PAI-1: plasminogenactivator inhibitor-1; SBE: smad binding element; SEAP: secreted alkaline phosphatase; TGF-β : Transforming Growth Factor-β; TMLC: transformed mink lung cell.

## Authors' contributions

IT carried out the experiments on measuring TGF-β from biological samples, coordinated and designed experiments, analyzed data and drafted the manuscript. KZ and HZ generated the stable MFB clones in a collaborative effort. EB carried out the sensitivity and specificity testing. TWC initiated the project and helped in writing the manuscript. All authors read and approved the final manuscript.
